# CRISPR-Cas9 Mediated Telomere Removal Leads to Mitochondrial Stress and Protein Aggregation

**DOI:** 10.3390/ijms18102093

**Published:** 2017-10-03

**Authors:** Hyojung Kim, Sangwoo Ham, Minkyung Jo, Gum Hwa Lee, Yun-Song Lee, Joo-Ho Shin, Yunjong Lee

**Affiliations:** 1 Division of Pharmacology, Department of Molecular Cell Biology, Sungkyunkwan University School of Medicine, Samsung Biomedical Research Institute, Suwon, Gyeonggi-do 440-746, Korea; hjung93@skku.edu (H.K.); ham89p12@skku.edu (S.H.); jmk4606@naver.com (M.J.); yslee@skku.edu (Y.-S.L.); 2 College of Pharmacy, Chosun University, Gwangju 501-759, Korea; gumhwalee@chosun.ac.kr; 3 Single Cell Network Research Center, Sungkyunkwan University School of Medicine, Suwon, Gyeonggi-do 440-746, Korea

**Keywords:** CRISPR-Cas9, telomere, aging, mitochondria, Parkinson’s disease

## Abstract

Aging is considered the major risk factor for neurodegenerative diseases including Parkinson’s disease (PD). Telomere shortening is associated with cellular senescence. In this regard, pharmacological or genetic inhibition of telomerase activity has been used to model cellular aging. Here, we employed CRISPR-Cas9 technology to instantly remove the telomere to induce aging in a neuroblastoma cell line. Expression of both Cas9 and guide RNA targeting telomere repeats ablated the telomere, leading to retardation of cell proliferation. Instant deletion of telomere in SH-SY5Y cells impaired mitochondrial function with diminished mitochondrial respiration and cell viability. Supporting the pathological relevance of cell aging by CRISPR-Cas9 mediated telomere removal, alterations were observed in the levels of PD-associated proteins including PTEN-induced putative kinase 1, peroxisome proliferator-activated receptor γ coactivator 1-α, nuclear respiratory factor 1, parkin, and aminoacyl tRNA synthetase complex interacting multifunctional protein 2. Significantly, α-synuclein expression in the background of telomere removal led to the enhancement of protein aggregation, suggesting positive feed-forward interaction between aging and PD pathogenesis. Collectively, our results demonstrate that CRISPR-Cas9 can be used to efficiently model cellular aging and PD.

## 1. Introduction

Aging is the most important risk factor for neurodegenerative disease including Parkinson’s disease (PD) [1]. In PD, progressive and age-dependent dopaminergic neuronal loss is thought to be mediated by the interplay among genes, environments, and aging [2]. Animal models of neurodegeneration have been characterized over their lifetime. Disease-linked gene mutation oftentimes results in significant neuronal demise only in elderly animals in several models of PD and Alzheimer’s disease [3,4]. Measures to slow aging, such as caloric restriction, can prevent neurodegeneration [5]. Although these studies identified several molecular alterations that could be associated with aging, in most cases, confounding and simultaneous interplay of natural aging and genetic background has made it difficult to dissect key pathogenic signaling pathways downstream of aging. To prevent the limitation of natural aging in application for neurodegenerative research, several aging cell/animal models have been developed by manipulating aging-associated gene alterations. These include telomere shortening, defective lamin processing, and defective mitochondrial DNA amplification [6].

Of these alterations, telomere shortening has been extensively investigated regarding its association with aging and aging model construction [7,8,9]. A telomere is a distinct structure at each end of chromosomes that is composed of tandem six nucleotide repeats of TTAGGG and a complex of shelterin proteins [8]. The repeat length of telomeres varies among different species and cell types. In somatic cells with no telomerase expression, telomeres shorten by 50–100 base pairs with each round of cell division [7,8,9]. Repetitive cell division results in naked telomere ends with excess telomere shortening [10]. This telomere erosion can be sensed by p53. The subsequent activation of p53 can lead to irreversible cell cycle arrest or apoptosis depending on the cellular context [10,11]. In addition to cell division, telomere shortening can also be induced by oxidative stress-mediated formation of 8-oxo-7,8-dihydro-2′-deoxyguanosine (8-oxodG) at the GGG triplet in telomere sequence, which can lead to telomere cleavage [12]. Since oxidative stress is implicated in pathogenic processes in neurodegeneration [13], telomere shortening seems to play a role in postmitotic neurons. Interestingly, rodent brains display telomere shortening as aging progresses [14].

Several approaches have sought to model telomere shortening as an aging process. Telomere shortening can be achieved by small interfering (si) RNA or chemical-mediated inhibition of telomerase enzyme (Tert) or RNA (Terc) components. Animal models of accelerated aging, which feature deletion of either *Tert* or *Terc*, have been developed [6,15]. These mouse models have been used to study the molecular mechanisms of aging and PD interaction. Dysfunction of mitochondria has been implicated in many neurodegenerative and aging-related disorder [16]. For instance, telomere shortening has been shown to induce mitochondrial dysfunction. Telomere shortening-induced DNA damage dissociates shelterin protein complex and exposes naked DNA ends which could be sensed by DNA damage sensors such as p53 [17]. Activated p53 in turn represses transcription of PGC-1α that is critical for maintenance of mitochondrial biogenesis and antioxidant defense in diverse tissues. Moreover, it has been shown that protein aggregation is facilitated by telomere shortening [18]. It is thought that abnormal microglia biology and cell aging influence α-synuclein protein aggregation. Although telomere shortened animal models have a unique advantage in investigating molecular interaction in aging process, the need for successive mating to induce sufficient telomere erosion limits their extensive use in neurodegeneration research.

Sequence specific DNA engineering has been made possible by development of nucleases including site-directed zinc finger nucleases (ZFNs), TAL effector nucleases (TALENs), and, more recently, endonuclease Cas9 that can be targeted to specific DNA loci via a single guide RNA (sgRNA) [19]. Depending on the presence of a protospacer adjacent motif sequence in the target DNA sequence, Cas9 can cleave double-stranded DNA. In this manner, clustered regulated interspaced short palindromic repeat (CRISPR) and CRISPR-associated (CRISPR-Cas9) system using expression of Cas9 and specifically designed sgRNA enables the efficient and easy manipulation of certain genes to study the functional outcomes of disease-associated genetic mutations [19]. CRISPR-Cas9 application is not limited to gene manipulation. A modified version of Cas9 with fluorescent reporter has been successfully targeted to repeated sequences including telomeres to label this distinct genomic structure [20].

In this study, we created a CRISPR-Cas9 system that specifically targets and removes telomere repeats. CRISRP-Cas9-mediated telomere removal led to mitochondrial dysfunction, cell growth arrest, and toxicity. Gene expression studies revealed alteration of PD-associated protein expression and α-synuclein solubility. This novel model of CRISPR-Cas9 aided telomere removal has potential value for PD and aging research.

## 2. Results

### 2.1. Efficient Telomere Removal by CRISPR-Cas9

**Figure 1:**
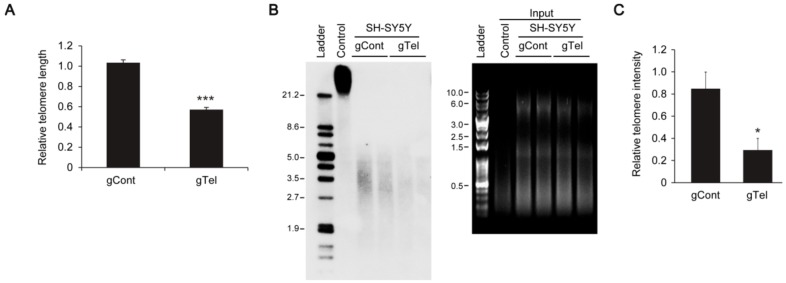
Reduction of telomere repeats by telomere targeting CRISPR-Cas9 in SH-SY5Y cells. (**A**) Quantification of telomere repeats in SH-SY5Y cells transiently transfected (48 h) with lentiCRISPR-gRNA-telomere (gTel) or mock plasmid (gCont) determined by real-time quantitative PCR using telomere specific primer sets. Relative telomere average length was normalized to the amounts of genomic locus *36B4u* (*n* = 3 per group); (**B**) Representative Southern blot images showing telomere restriction fragments obtained from the same amounts of genomic DNA extracted from SH-SY5Y cells transfected with gTel or gCont (48 h). Telomere fragments were detected by telomere sequence specific probe (left panel). Hinf I/Rsa I digested genomic DNA separated on agarose gel and visualized by RedSafe nucleic acid staining as loading control for Southern blot (right panel); (**C**) Quantification of telomere intensities determined by Southern blot experiments normalized by digested genomic DNA loading amounts (*n* = 3 per group). Quantified data are expressed as mean ± SEM. Statistical significance was determined by unpaired two-tailed Student’s *t* test, * *p* < 0.05, and *** *p* < 0.001.

It has been reported that Cas9 variant with no endonuclease activity can be efficiently targeted to telomere structure using sgRNA. To determine whether the CRISPR-Cas9 system can cleave the telomere sequence, we cloned sgRNA sequence targeting telomere repeats into a lentiCRISPR-v2 construct that expresses both Cas9 endonuclease and sgRNA (lentiCRISPR-sgRNA-telomere). The lentiCRISPR-sgRNA-telomere was introduced into SH-SY5Y cells by transient transfection. Relative amounts of telomere repeats were monitored by real-time quantitative PCR (RTQ PCR) using telomere specific primers as described previously [21]. Expression of Cas9 and sgRNA-telomere led to an approximate 50% reduction of telomere amounts when compared to mock DNA transfection (Figure 1A). The lengths of telomere restriction fragments (TRF) were also determined by Southern blot analysis using telomere specific probe incubation. Interestingly, we did not observe shortening of TRF length (Figure 1B). However, there was over 70% decline in TRF band intensity, indicating removal of intact telomeres. The transcription of Tert was not influenced by CRISPR-Cas9 induced telomere removal in SH-SY5Y cells (Figure S1).

### 2.2. Limited Cell Proliferation and Mitochondrial Dysfunction by CRISPR-Cas9-Mediated Telomere Removal

**Figure 2:**
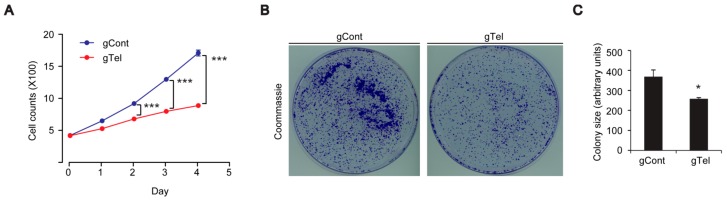
Limited replicative potential of SH-SY5Y cells with telomere removal by CRISPR-Cas9. (**A**) Cell growth assay for SH-SY5Y cells transfected with gTel or gCont and determined by trypan blue cell counting at the indicated days (*n* = 7 per group); (**B**) Representative image of SH-SY5Y cells stained with coomassie blue from colony formation assay; (**C**) Quantification of average colony size for coomassie stained SH-SY5Y cell colonies per each group (*n* = 3 plates per group). Quantified data are expressed as mean ± SEM. Statistical significance was determined by unpaired two-tailed Student’s *t* test or ANOVA test with Tukey post-hoc analysis, * *p* < 0.05, and *** *p* < 0.001.

Following successful removal of telomere repeats by CRIPSR-Cas9 expression, we sought to determine whether this type of telomere removal can recapitulate previously characterized phenotypes of cell aging models, including cell growth arrest and mitochondrial dysfunction. SH-SY5Y cells were transfected with lentiCRISPR-sgRNA-telomere or control mock plasmid (Day 0), and, from the following day (Day 1), they were subjected to trypan blue staining and cell enumeration until 4 days after transfection. Cell counting revealed the decreased cell proliferation following telomere removal by CRIPSR-Cas9 expression (Figure 2A). Colony forming ability was also determined by coomassie staining for SH-SY5Y cells plated onto culture dishes and cultured for 7 days. A significant reduction of average colony size was evident in plates with lentiCRISPR-sgRNA-telomere transfection (Figure 2B,C) indicating limited cell replicative potential.

**Figure 3:**
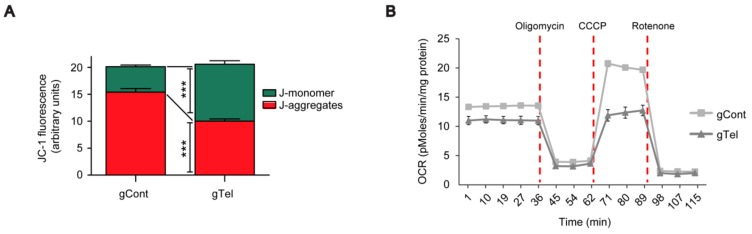

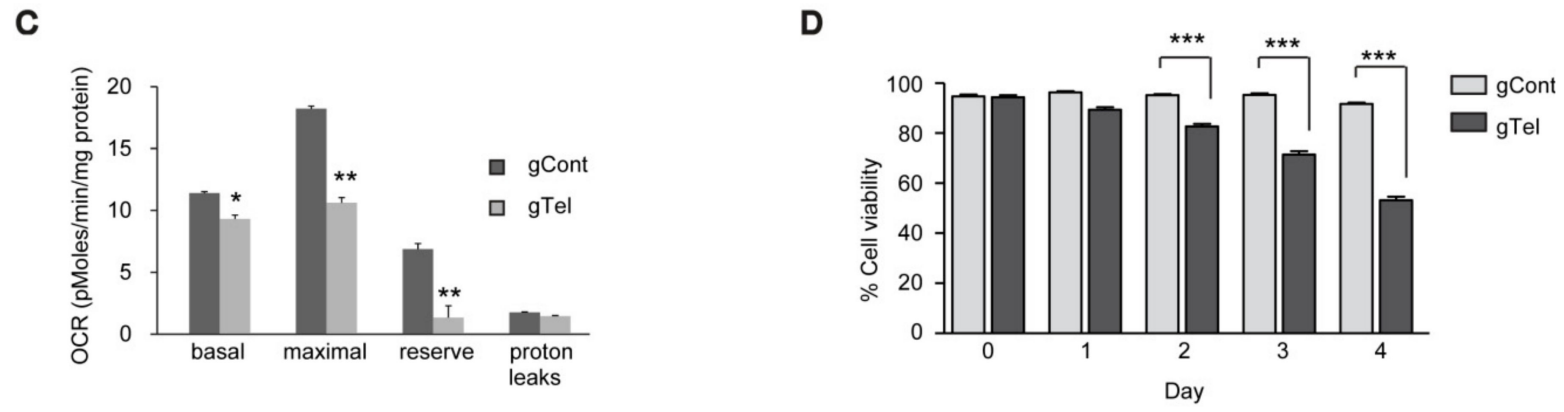
Mitochondrial dysfunction induced by CRISPR-Cas9 mediated telomere removal. (**A**) Quantification of red and green fluorescence intensities for each SH-SY5Y cell culture plate stained with JC-1 dye from the indicated experimental groups (*n* = 7 wells per group); (**B**) Mitochondrial stress testing of SH-SY5Y cells transfected with gTel or gCont determined by Seahorse oxygen consumption rate measurements; (**C**) Telomere removal by CRISPR-Cas9 caused an approximate 18% decrease in basal respiration and a 42% decrease in maximal respiration. CRISPR-ca9 mediated telomere removal reduced mitochondrial reserve capacity by 81% (*n* = 5 assays per group); (**D**) Cell viability assessment showing gradually increased cell toxicity after expression of telomere targeting CRISPR-Cas9 in SH-SY5Y cells determined by the Trypan blue exclusion assay at the indicated days following transient transfection (*n* = 7). Quantified data are expressed as mean ± SEM. Statistical significance was determined by unpaired two-tailed Student’s *t* test or ANOVA test with Tukey post-hoc analysis, * *p* < 0.05, ** *p* < 0.01, and *** *p* < 0.001.

Next, mitochondrial function was assessed by JC-1 dye, which emits red fluorescence for depolarized healthy mitochondria (JC-1 aggregates) and green fluorescence for damaged mitochondria with dissipated mitochondria potential (JC-1 monomer) [22]. SH-SY5Y cells were transfected with gTel and gCont. The following day, they were plated in well of 96-well plates for subsequent 48 h culture in complete medium and JC-1 fluorescence plate reading. A relative increase of mitochondrial damage was evident in telomere free SH-SY5Y cells with CRISPR-Cas9 application as observed by increased JC-1 monomer fluorescence as compared to the concomitant reduction of JC-1 aggregate fluorescence (Figure 3A). In a similar fashion, HEK-293T cells transiently transfected with CRISPR-Cas9 displayed telomere shortening (Figure S2A) and disruption of mitochondrial potential (Figure S2B), suggesting potential application of this tool in diverse cell lines. Mitochondrial respiration in SH-SY5Y cells was also monitored by measurement of oxygen consumption rates in mitochondrial stress paradigm. There was an 18% reduction in basal respiration, 42% reduction in carbonyl cyanide m-chlorophenyl hydrazine (CCCP)-induced maximal respiration, and 81% reduction in spare respiratory capacity (Figure 3B,C). In agreement with dysfunctional mitochondria, CRISPR-Cas9-mediated telomere removal in SH-SY5Y cells led to the gradual decrease of cellular viability, which reached almost 50% cell death at Day 4 using the trypan blue exclusion assay (Figure 3D). Reduction of cell viability (approximate 40% decrease) by CRISPR-Cas9-mediated telomere removal was also observed by Cell Counting Kit-8 (CCK8)-based assay (Figure S3). These results indicated that telomere removal by CRISPR-Cas9 can recapitulate key aging associated features in neuroblastoma cell lines within several days.

### 2.3. Alteration of PD Associated Protein Regulation in Telomere-Less SH-SY5Y Cells

**Figure 4:**
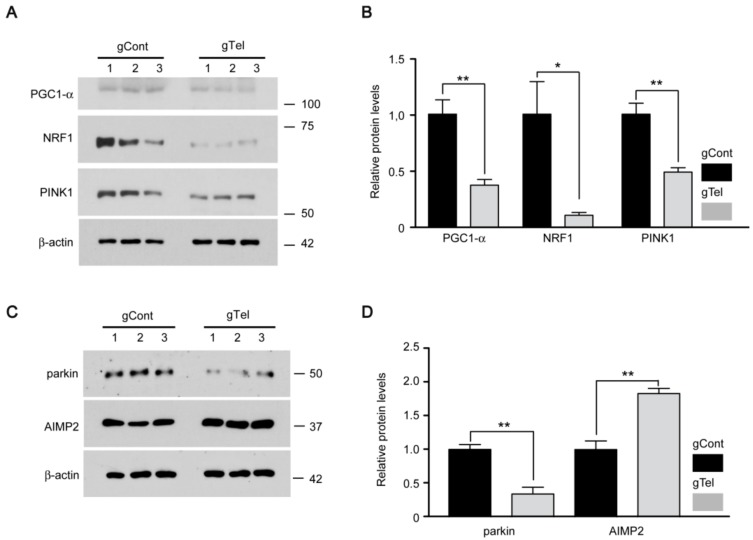
Alteration of mitochondria and PD associated proteins in SH-SY5Y cells with telomere removal by CRISPR-Cas9. (**A**) Representative Western blot of PGC-1α, NRF1, and PINK1 in SH-SY5Y cells transfected with either gTel or gCont (72 h). β-actin served as a loading control; (**B**) Quantification of relative protein levels normalized to β-actin for the indicated experimental groups (*n* = 3 per group); (**C**) Representative Western blot of parkin and AIMP2 in SH-SY5Y cells transfected with either gTel or gCont (72 h). β-actin served as a loading control; (**D**) Quantification of relative protein levels normalized to β-actin for the indicated experimental groups (*n* = 3 per group). Quantified data are expressed as mean ± SEM. Statistical significance was determined by unpaired two-tailed Student’s *t* test, * *p* < 0.05, and ** *p* < 0.01.

Mitochondrial dysfunction has been implicated in PD etiology [2,16]. Several PD genes and their interacting partners have been shown to be involved in mitochondrial regulation [2]. Especially, peroxisome proliferator-activated receptor γ coactivator 1-α (PGC-1α), the master regulator of mitochondrial biogenesis, and its downstream target genes are downregulated in postmortem PD brains as well as PD animal models, indicating their importance in PD pathogenesis [23]. To determine whether the PGC-1α pathway is disrupted in our CRISPR-Cas9 model of telomere removal, expression levels of PGC-1α and its major target gene nuclear respiratory factor 1 (*NRF1*) were monitored in SH-SY5Y cells expressing Cas9 and sgRNA-telomere by Western blot. An approximate 50% and 80% decline in expression levels of PGC-1α and NRF1 by telomere removal was evident, respectively, compared to control transfection (Figure 4A,B). We also examined the expression of PD-linked mitochondrial serine/threonine kinase PTEN-induced putative kinase 1 (*PINK1*), which is involved in antioxidant defense and mitochondrial quality control by mitophagy and biogenesis [24]. Interestingly, PINK1 expression was also decreased by about 40% following telomere degradation by CRISPR-Cas9 (Figure 4A,B). Since DNA damage sensors such as p53 and poly (ADP-ribose) polymerase 1 (PARP1) are involved in mitochondrial regulation following telomere deficits [25,26,27], we determined whether expression of these DNA damage sensors was altered in SH-SY5Y cells following telomere removal. P53 was elevated by more than three folds after CRISPR-Cas9 mediated telomere removal (Figure S4A,B). Moreover, marked enhancement of self-PARsylation of PARP1 occurred in SH-SY5Y cells in response to telomere removal (Figure S4A,B), indicating hyperactivation of PARP1. These results suggest that activation of DNA damage response and dysregulation of key proteins in mitochondrial biogenesis and quality control may be involved in the mitochondrial function defects downstream of telomere removal by CRISPR-Cas9.

Subsequent experiments examined cell death pathways involved in dopaminergic neurodegeneration in PD. Parkin is an E3 ubiquitin protein ligase whose mutation or inactivation contributes to PD pathogenesis [28]. We have reported that the accumulation of the pathogenic parkin substrate, aminoacyl tRNA synthetase complex interacting multifunctional protein 2 (AIMP2), leads to poly (ADP-ribose) polymerase 1 (PARP1) activation and a distinct cell death pathway called parthanatos [29]. To understand the molecular pathways involved in the death of SH-SY5Y cells following CRISPR-Cas9 mediated telomere removal, we assessed expression of parkin and its substrate, AIMP2. Parkin expression decreased by 65% as compared to control transfection, while AIMP2 levels were elevated almost two-fold (Figure 4C,D).

**Figure 5:**
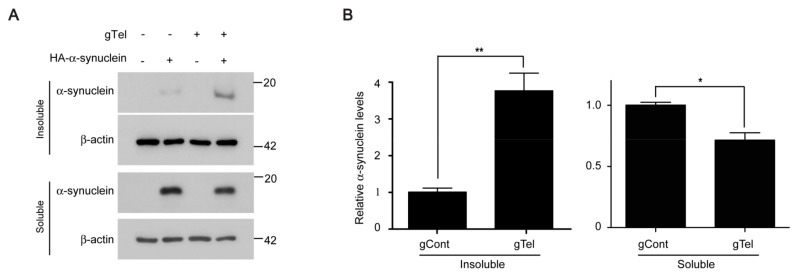
Redistribution of α-synuclein into insoluble fraction by CRISPR-Cas9 mediated telomere removal (**A**) Representative Western blots of α-synuclein expression in 1% Triton X soluble and insoluble fractions from SH-SY5Y cells transfected with the indicated combinations of HA-α-synuclein, gTel, and gCont constructs (72 h); (**B**) Quantification of relative amounts of α-synuclein expression in insoluble or soluble fractions normalized to β-actin internal loading control (*n* = 3 per group). Quantified data are expressed as mean ± SEM. Statistical significance was determined by unpaired two-tailed Student’s *t* test, * *p* < 0.05, and ** *p* < 0.01.

It has been suggested that aging and telomere shortening influence α-synuclein aggregation, a hallmark of PD pathogenesis [2]. To determine whether the CRISPR-Cas9 system of telomere removal influences α-synuclein aggregation, SH-SY5Y cells with gTel or gCont transfection were subsequently transfected with hemagglutinin tagged (HA)-α-synuclein constructs. α-Synuclein aggregation was indirectly examined by separation of protein lysates into 1% Triton X soluble and insoluble fractions followed by Western blot analysis of α-synuclein distribution. gTel transfection resulted in more than three-fold increase of α-synuclein distribution into insoluble fractions, whereas gCont transfection failed to induce any observable α-synuclein insolubility (Figure 5A,B). Interestingly, α-synuclein redistribution into insoluble fraction by telomere removal by CRISPR-Cas9 was accompanied by about a 25% reduction of α-synuclein in the soluble fraction (Figure 5A,B).

## 3. Discussion

This study provides the first demonstration that efficient telomere removal is possible by CRISPR-Cas9 system using telomere targeting sgRNA. Telomere shortening has been accomplished by the siRNA-mediated inhibition of telomerase or using chemical inhibitors [6,11,17,30,31]. The weakness of these methods is that subsequent cell proliferation is required to attain the substantial loss of telomere repeats. Our CRISPR-Cas9 application allows immediate telomere removal as early as two days following transfection without the need for continuous cell proliferation and passage. This immediate telomere removal could be beneficial to understand molecular pathways that are directly associated with telomere shortening, since telomerase inhibition and repeated cell division can cause adaptation of cells. Moreover, telomerase has been shown to exert telomere independent functions [32]. Our CRIPSR-Cas9 telomere removal could be used to efficiently study functional and molecular outcomes downstream of telomere dysfunction in several cell types. Especially, brains are composed of postmitotic neurons as well as dividing glial cells. Direct targeting and cleavage of telomere in neuronal population could be possible by CRISPR-Cas9, while telomerase inhibition cannot accomplish telomere shortening in postmitotic cell types.

It appears that CRISPR-Cas9 mediated telomere removal is quite robust, since we observed elimination of TRF intensities in Southern blots rather than reduction of TRF length. Given the approximate 60% transfection efficiency in SH-SY5Y cells, it is possible that once lentiCRISPR-sgRNA-Telomere is introduced in SH-SY5Y cells almost complete degradation of telomere repeats is induced, making detection of partially shortened TRF difficult. CRISPR-cas9-induced telomere removal could be further validated in cell and chromosome resolution by using Fluorescence in situ hybridization (FISH) technology.

Mitochondrial dysfunction has been implicated in diverse human disease including PD. The mitochondrial toxin, 1-methyl-4-phenyl-1,2,3,6-tetrahydropyridine (MPTP), can recapitulate many of key pathogenic alterations of PD in human and animals [33]. In addition, defective mitochondrial biogenesis has been observed in PD animal models [23]. The master regulator of mitochondria biogenesis is PGC-1α. Its expression is repressed downstream of parkin inactivation via accumulation of the parkin-interacting substrate (PARIS) [23]. Interestingly, PGC-1α null dopaminergic neurons display enhanced susceptibility to MPTP-induced neurodegeneration, indicating the interaction of mitochondria biogenesis and environmental risk [34]. PGC-1α also has shown active interaction with aging process in a report using telomerase null mice (either Tert or Terc gene deletion). Telomere dysfunction induced PGC-1α repression and impaired mitochondrial biogenesis and function with increased reactive oxygen species in liver or heart tissues via p53 activation [17]. This study agrees with our results showing mitochondrial dysfunction and decreased expression of PGC-1α/NRF1 downstream of direct telomere removal by CRISPR-Cas9. DNA damage by telomere erosion can be sensed by diverse protein sensors which include ATM/CHK2, p53 [27], and PARP1 [26]. Significantly p53 and PARP1 were activated in response to CRISPR-Cas9-mediated telomere removal. Since p53 and PARP1 hyperactivation has been implicated in neurodegeneration of PD [29,35], these DNA damage sensors might have contributed to PGC-1α repression, and mitochondrial dysfunction following telomere removal by CRISPR-Cas9. It would be instructive to further characterize mitochondrial biogenesis and functions in different organs by applying lentiCRISPR-sgRNA-telomere using adenoassociated virus delivery.

Several PD linked genes including *α-synuclein*, *DJ-1*, *PINK1*, and *parkin* influence mitochondria regulation. Especially, PINK1 and parkin corroborate to regulate mitochondrial quality control by selectively tagging damaged mitochondria and targeting them for autophagy-mediated clearance. Defective PINK1 or parkin results in defective structure and function of mitochondria [36]. The present observations suggest that telomere dysfunction could influence mitochondrial quality control because PINK1 and parkin are downregulated. Our cell model of telomere defect can be used as platform for studies of the potential involvement of mitophagy and its underlying molecular mechanisms.

PD is the most common neurodegenerative movement disorder for which there is no effective treatment to halt or delay progression of age dependent dopaminergic neuron loss. The lack of therapeutic options is largely due to the incomplete understanding of molecular mechanisms of complex interaction among aging, genetic background, and environments. Moreover, most PD animal models have failed to recapitulate progressive dopaminergic cell loss in a short time window [4]. In this respect, the combination of PD-linked animal models with aging models could accelerate the progression of disease pathologies, and thereby facilitate PD research. In this study, we found that CRIPSR-Cas9-mediated telomere removal recapitulated several key cellular and molecular features that have been reported for previous aging models of telomere dysfunction. Interestingly, the induction of telomere removal by CRISPR-Cas9 in only several days was sufficient to produce α-synuclein insolubility. α-Synuclein aggregation is a process that requires a long incubation time. Regular α-synuclein overexpression fails to produce α-synuclein aggregation [37]. Moreover, transgenic mouse expression α-synuclein in brains only displays α-synuclein aggregation at advanced ages [4]. The combination of our CRIPSR-Cas9 telomere removal model with current PD animal models could potentially accelerate PD related pathology. Of note, a recent report showed that the combination of α-synuclein transgenic mice with third-generation Terc null mice resulted in enhanced synucleinopathy and impaired microglia response [18]. Application and validation of virus-mediated delivery of gTelomere CRISPR-Cas9 into mouse brains will address molecular mechanisms of neuronal and glial regulation controlled by telomere function.

## 4. Materials and Methods

### 4.1. Reagents and Antibodies

Primary antibodies were mouse antibody to α-synuclein (cat# 610787, 1:3000, BD Transduction), mouse antibody to parkin (cat# 4211, 1:3000, Cell Signaling Technology, Danvers, MA, USA), mouse antibody to PGC-1α (cat# ST1202, 1:1000, Millipore, Billerica, MA, USA) rabbit antibody to PINK1 (cat# 100-494, 1:2000, Novus Biologicals, Littleton, CO, USA), rabbit antibody to AIMP2 (cat# 10424-1-AP, 1:3000, Proteintech Group, Manchester, UK), mouse antibody to p53 (cat# SC-126, 1:1000, Santa Cruz Biotechnology, Dallas, TX, USA), mouse antibody to PAR (cat# 4335-MC-100, 1:3000, Trevigen, Gaithersburg, MD, USA), and rabbit antibody to PARP (cat# 9542S, 1:3000, Cell Signaling Technology). Secondary antibodies were horseradish peroxidase (HRP)-conjugated mouse antibody against β-actin (AC15, 1:10,000, Sigma-Aldrich, St. Louis, MO, USA), HRP-conjugated sheep antibody against mouse IgG (cat# RPN4301, 1:5000, GE Healthcare, Little Chalfont, UK), and HRP-conjugated donkey antibody against rabbit IgG (cat# RPN4101, 1:5000, GE Healthcare).

### 4.2. Cell Culture and Transfection

Human neuroblastoma SH-SY5Y cells (ATCC) and human embryonic kidney (HEK)-293T cells were grown in Dulbecco’s Modified Eagle’s Medium (DMEM) containing 10% (*v*/*v*) fetal bovine serum (FBS) and antibiotics. Cells were cultured at 37 °C in a humidified incubator supplied with 5% CO_2_/95% air. For transient transfection, cells were transfected with indicated plasmid constructs using X-tremeGENE HP transfection reagents (Roche, Basel, Switzerland) according to the manufacturer’s instructions. For subsequent analysis following CRISPR-Cas9 mediated telomere removal, SH-SY5Y cells or HEK-293T cells were replated on appropriate culture plates 24 h after lentiCRISPR-gRNA (telomere) transfection.

### 4.3. Plasmids

LentiCRISPR-v2 construct was purchased from Addgene. Oligonucleotides were synthesized by Cosmo Genetech. LentiCRISPR-gRNA to human telomere repeats was constructed by ligating gRNA oligonucleotides into BsmBI restriction site of pLenti-CRISPR-v2 plasmid (Addgene). The gRNA targeting sequence for telomere sequence was (F: CACCGTAGGGTTAGGGTTAGGGTTA; R: AAACTAACCCTAACCCTAACCCTAC). The HA-α-synuclein construct was described previously [38].

### 4.4. Telomere Quantification by Real-Time PCR

Total genomic DNA was extracted from cells by lysing cells in DirectPCR lysis reagent (102-T; Viagen) supplemented with proteinase K (Roche) followed by DNA purification using phenol:chloroform:isoamyl alcohol (25:24:1; Sigma-Aldrich). Cycle threshold (*C*_t_) values of each DNA loci were obtained from SYBR Green based real-time PCR using QuantStudio 6 flex Real-Time PCR System (Applied Biosystems, Foster City, CA, USA). The thermal cycling protocol for both amplicons began with a 95 °C incubation for 10 min to activate the AmpliTaq Gold DNA polymerase. For telomere PCR, 40 cycles of 95 °C for 15 s, 54 °C for 2 min were carried out with fluorescence reading at each amplification step. For *36B4u* PCR, 40 cycles of 95 °C for 15 s, 58 °C for 1 min were carried out with fluorescence reading at each amplification step. Relative amounts of telomere repeats [21] were calculated by ΔΔ*C*_t_ method using *36B4u* as an internal loading control as previously described [39]. SYBR Green PCR master mix (Cat# 4309155, Applied Biosystems) was used according to the manufacturer’s instructions. The following primers were used for real-time PCR amplification: Telomere-F (GGTTTTTGAGGGTGAGGGTGAGGGTGAGGGTGAGGGT), Telomere-R (TCCCGACTATCCCTATCCCTATCCCTATCCCTATCCCTA), 36B4u-F (CAGCAAGTGGGAAGGTGTAATCC), 36B4u-R (CCCATTCTATCATCAACGGGTACAA). The final telomere primer concentrations were: telomere-F, 270 nM; telomere-R, 900 nM. The final *36B4u* (single copy gene) primer concentrations were: 36B4u-F, 300 nM; 36B4u-R, 500 nM.

### 4.5. Telomere Assay

SH-SY5Y cells transiently transfected with lentiCRISPR-gRNA to telomere or mock-infected cells were used for the TeloTAGGG telomere length assay (Roche) following the manufacturer’s instructions. Briefly, genomic DNA was purified by conventional phenol-chloroform extraction (Ultra-Pure phenol:chloroform:isoamyl alcohol, 25:24:1 (*v*/*v*); ThermoFisher Scientific). Purified genomic DNA was digested with restriction enzymes supplied in the kit and DNA fragments were separated by gel electrophoresis and visualized under UV after staining (RedSafe Nucleic Acid Staining Solution, JH Science). gDNA restriction fragments were transferred to nylon membrane. Telomere restriction fragments (TRF) were visualized by hybridization with digoxingenin-labeled probe specific for telomeric repeats.

### 4.6. Real-Time Quantitative PCR

Total RNA was extracted with QIAzol Lysis Reagent (cat# 79306, QIAGEN, Hilden, Germany) from SH-SY5Y cells two days after transient transfection with gTel or gControl, and then treated with DNase I to eliminate trace DNA contamination. cDNA was synthesized from total RNA (1.5 μg) using a first-strand cDNA synthesis kit (iScript cDNA synthesis kit, Bio-Rad, Hercules, CA, USA). The relative quantities of mRNA expression were analyzed using real-time PCR (QuantStudio 6 flex Real-Time PCR System, Applied Biosystems). SYBR Green PCR master mix (Cat# 4309155, Applied Biosystems) was used according to the manufacturer’s instructions. The relative mRNA expression levels of target genes were calculated by the ΔΔ*C*_t_ method [39] using GAPDH as an internal loading control. The primer sequences for real-time gene amplification are as follows:

hGAPDH: F-AAACCCATCACCATCTTCCAG, R-AGGGGCCATCCACAGTCTTCT;
hTert: F-CCGATTGTGAACATGGACTACG, R-CACGCTGAACAGTGCCTTC.

Primers seqeunce for Tert gene has been described previously [40].

### 4.7. Colony Formation Assay

Twenty-four hours after transient transfection, SH-SY5Y cells were seeded with 2500 cells/well in 10 cm-diameter cell culture dishes. After 7 days of incubation, cells were washed with phosphate buffered saline (PBS), fixed with 3.7% formaldehyde and 70% ethanol, and stained with 0.05% Coomassie blue. Images were scanned and colony size were analyzed by ImageJ software (https://imagej.nih.gov/ij/, the National Institutes of Health).

### 4.8. JC-1 Mitochondrial Functional Assay

To assess mitochondrial membrane potential, the lipophilic cationic JC-1 dye was used as part of the JC-1 mitochondrial membrane potential assay kit (Cayman Chemical, Ann Arbor, MI, USA) following the manufacturer’s instructions. Briefly, 24 h following transient transfection of lentiCRISPR-gRNA for telomere or mock plasmids, SH-SY5Y or HEK-293T cells were plated in wells of a 96-well plate at a density of 5 × 10^5^ cells/well. After 48 h of cell culture in the complete medium, cells were stained with JC-1 (10 uL of the JC-1 staining solution per 100 uL of culture medium) for 20 min in the cell culture incubator and washed twice with assay buffer. JC-1 fluorescence were measured by using a SYNERGYneo microplate reader (Bio-Tek, Winooski, VT, USA) with excitation and emission wavelengths of 535 and 595 nm, respectively, emission for healthy mitochondria and 485 and 535 nm, respectively, for unhealthy mitochondria. The relative healthiness of mitochondria membrane potential was expressed as ratio of fluorescence intensities obtained at two different excitation/emission settings.

### 4.9. Oxygen Consumption

Oxygen consumption rates (OCRs) for SHSY-5Y cells were assayed 48 h post-transfection using a standard mitochondrial stress test paradigm using an XF-24 analyzer (Seahorse Bioscience, North Billerica, MA, USA). Cells were washed once with assay medium (DMEM containing 10 mM glucose, 1 mM pyruvate, and 2 mM glutamine) before adding 675 μL of assay media to each well and incubating in a non-CO_2_, 37 °C incubator for 30–60 min. Oxygen consumption rate (OCR) was measured using a 1-min mix, 1-min wait, and a 2-min measurement cycle. Injection ports contained: (i) dimethylsulfoxide (0.002% *v*/*v*); (ii) oligomycin (1.25 μg/mL); (iii) carbonyl cyanide *m*-chlorophenyl hydrazine (CCCP, 1.25 μM); and (iv) rotenone (1 μM). For SHSY5Y cells, antimycin-A (1 μM) was added to the final injection port. OCR values were normalized to total protein, determined for each well by bicinchoninic acid (BCA) assay, and rotenone insensitive (nonmitochondrial) oxygen consumption was subtracted from all values. Basal respiration was calculated using the mean of the three OCR measurements before the first injection and maximal respiration was calculated as the mean of three OCR measurement cycles after CCCP injection. Reserve capacity was calculated by subtracting the basal OCR values from CCCP-induced maximal OCR. Proton leaks were calculated by subtracting rotenone-insensitive OCR values from basal OCR.

### 4.10. Cell Viability and Growth Assay

SH-SY5Y cells were plated in wells of 6-well plates at a seeding density of 0.5 × 10^6^ cells per well. Following transient transfection with indicated constructs, SH-SY5Y cells were plated onto complete medium the next day and grown. On the indicated days, SH-SY5Y cells were trypsinized, yielding single cell suspensions. Cells were harvested and washed twice with PBS before resuspension in serum-free DMEM. Resuspended cells were mixed with an equal volume of 0.4% trypan blue (*w*/*v*) and incubated at room temperature for 2 min. Live and dead cells were counted using an EVE™ automatic cell counter (NanoEnTek, Seoul, Korea). Cell Counting Kit-8 (CCK8, DOJINDO Molecular Technologies, Rockville, MD, USA) was also used to assess viability of SH-SY5Y cells or primary cultured neurons following the manufacturer’s instructions.

### 4.11. Western Blot Analysis

For Triton X-100-soluble and -insoluble fraction separation, SH-SY5Y cells were harvested and processed into nonionic detergent-soluble and detergent-insoluble fractions in lysis buffer containing PBS, 1% Triton X-100, phosphatase inhibitor cocktail II and III (Sigma-Aldrich), and a complete protease inhibitor mixture. The lysates were centrifuged at 100,000× *g* for 20 min at 4 °C. The resulting pellet and supernatant (S1, soluble) fractions were collected. The pellet was washed once in lysis buffer containing nonionic detergent (1% Triton X-100) and solubilized in lysis buffer containing 1% sodium dodecyl sulfate (SDS) and 0.5% sodium deoxycholate. The homogenate was centrifuged and the resulting supernatant (nonionic detergent-insoluble) was collected.

For total lysates, cells were harvested, washed twice with PBS, and lysed with Pierce RIPA buffer (150 mM NaCl, 50 mM Tris, pH 8.0, 1% NP40, 1% SDS, and 0.5% sodium deoxycholate, and a protease inhibitor mixture; ThermoScientific, Waltham, MA, USA) for 30 min on ice. Following this, cell lysates were prepared by centrifugation (22,250× *g* at 4 °C for 20 min). Protein concentration was determined using the Pierce™ BCA protein assay kit (ThermoScientific). Equal amounts of protein (10–20 μg) were resolved on 8–16% sodium dodecyl (lauryl) sulfate-polyacrylamide gel electrophoresis (SDS-PAGE) and transferred to a nitrocellulose membrane. After washing with TBST (Tris-buffer solution-Tween 20; 10 mM Tris-HCl (pH 7.6), 150 mM NaCl, and 0.05% Tween-20), membranes were blocked with 5% skim milk for 1 h and incubated with an appropriate primary antibody at the dilution recommended by the supplier. The membrane was then washed and primary antibodies were detected with HRP-conjugated secondary antibody. Immunoblot signals were visualized using a chemiluminescence kit (Pierce). Densitometric analyses of immunoreactive bands were performed using ImageJ software. The ratio between treated and control samples was calculated for each individual experiment and expressed relative to the control.

### 4.12. Statistics

Quantitative data are presented as mean ± SEM. Statistical significance was assessed via an unpaired two-tailed Student’s *t* test for comparison of two groups (control and test) or an ANOVA test and Student Newman–Keuls post-hoc analysis for comparison among multiple groups of more than three as indicated in each figure legend. *p* Values lower than 0.05 were considered to indicate significant difference among groups.

## Supplementary Materials

Supplementary materials can be found at www.mdpi.com/1422-0067/18/10/2093/s1.

## Abbreviations

CRISPR-Cas9	Clustered regulated interspaced short palindromic repeat and CRISPR-associated (Cas)
PD	Parkinson’s disease
PGC-1α	Peroxisome proliferator-activated receptor γ coactivator 1-α
NRF1	Nuclear Respiratory Factor 1
PINK1	PTEN-induced putative kinase 1
AIMP2	Aminoacyl tRNA synthetase complex interacting multifunctional protein 2
