# Supplementary material for: CRISPR-Cas9 Mediated Telomere Removal Leads to Mitochondrial Stress and Protein Aggregation
Source: Int J Mol Sci. 2017 Oct 3;18(10):2093. doi: 10.3390/ijms18102093 (PMC5666775; doi:10.3390/ijms18102093)
Supplement: Supplementary file 1 [file ijms-18-02093-s001.pdf]

Supplemental information

CRISPR-cas9 mediated telomere removal leads to mitochondrial stress and protein aggregation

Hyojung Kim, Sangwoo Ham, Minkyung Jo, Gum Hwa Lee, Yun-Song Lee, Joo-Ho Shin, and Yunjong Lee

Supplementary Figures

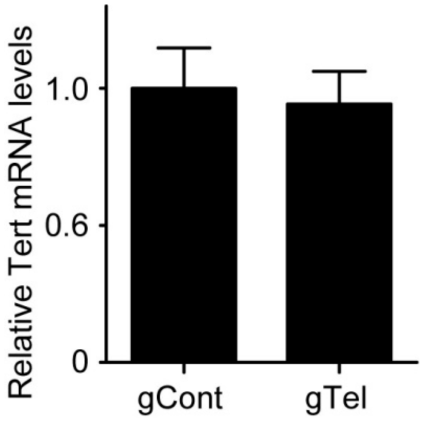

Figure S1. Quantification of relative Tert mRNA levels in SH-SY5Y cells determined 2 days after transient transfection with gTel or control by real-time quantitative PCR (n = 3).

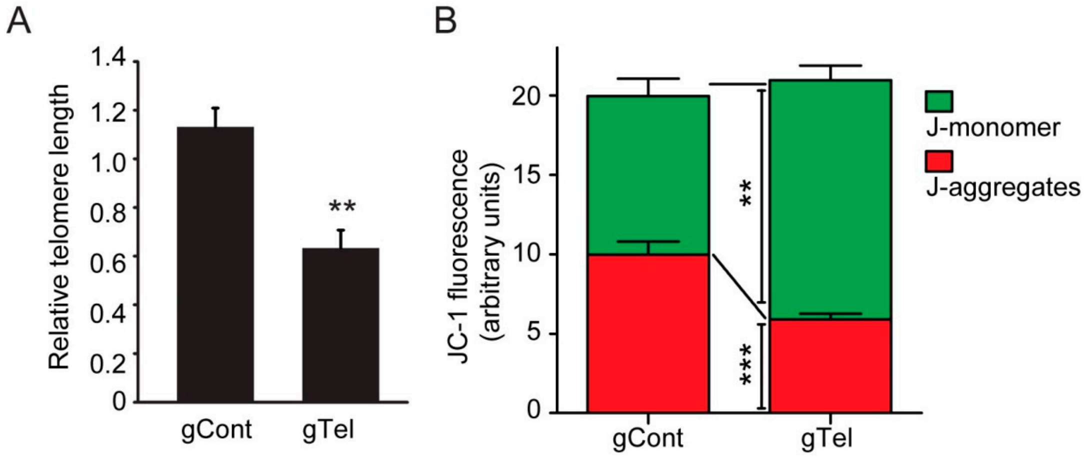

Figure S2. (**A**) Quantification of telomere repeats in HEK-293T cells transiently transfected (48 h) with lentiCRISPR-gRNA-telomere (gTel) or mock plasmid (gCont) determined by real-time quantitative PCR using telomere specific primer sets. Relative telomere average length was normalized to the amounts of genomic locus 36B4u (n = 3 per group). (**B**) Quantification of red and green fluorescence intensities for each HEK-293T cell culture plate stained with JC-1 dye from the indicated experimental groups (n = 8 wells per group). Statistical significance was determined by unpaired two-tailed Student's t test, **$p< 0.01$, and ***$p < 0.001$.

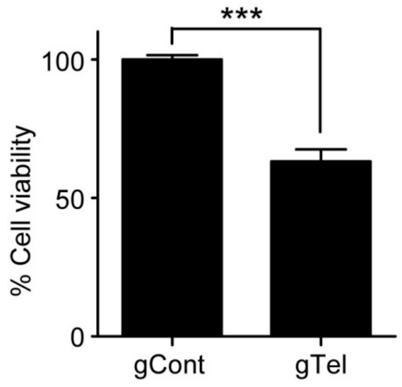

Figure S3. (**A**) Cell Counting Kit-8 (CCK8) viability assay demonstrating that lentiCRISPR-gRNA-telomere (gTel) expression reduces cell survival (*n* = 8 per group). SH-SY5Y cells were transfected with constructs driving the expression of cas9 and guide RNA for telomere or control for three days followed by CCK8 viability assay. Statistical significance was determined by unpaired two-tailed Student's *t* test, ***p < 0.001.

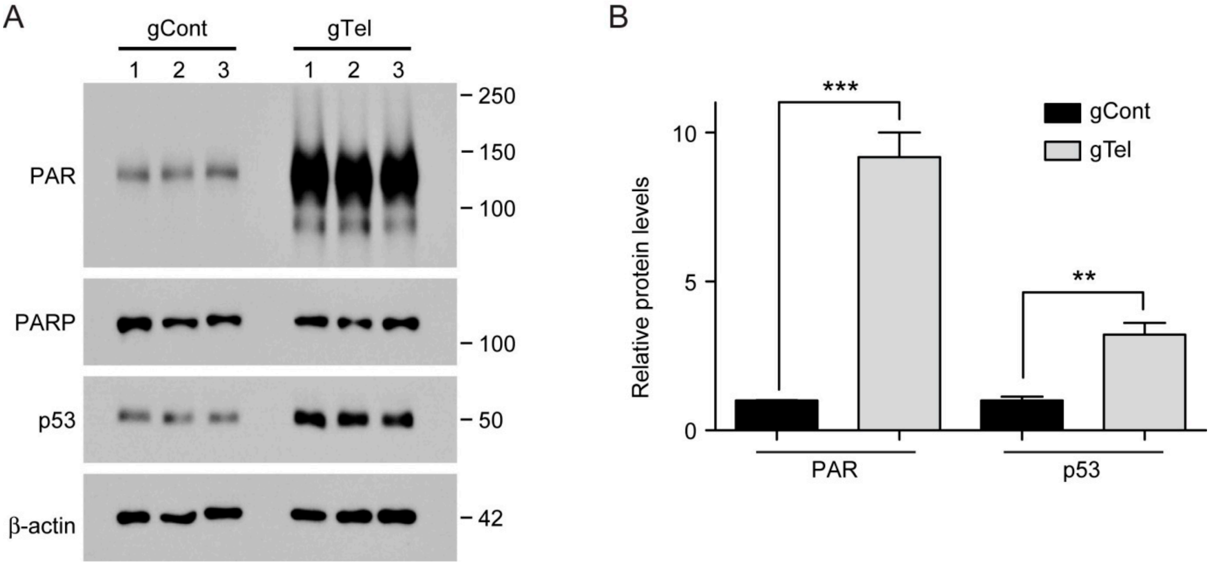

Figure S4. (**A**) Representative western blot of PAR-conjugated proteins, PARP, and p53 in SH-SY5Y cells transfected with either gTel or gCont (72 h). β-actin served as a loading control. (**B**) Quantification of relative protein levels normalized to β-actin for the indicated experimental groups (*n* = 3 per group). Quantified data are expressed as mean ± SEM. Statistical significance was determined by unpaired two-tailed Student's *t* test, **p< 0.01, and ***p < 0.001.
